# Cerebral autoregulation assessed by near-infrared spectroscopy: validation using transcranial Doppler in patients with controlled hypertension, cognitive impairment and controls

**DOI:** 10.1007/s00421-021-04681-w

**Published:** 2021-04-16

**Authors:** Arjen Mol, Carel G. M. Meskers, Marit L. Sanders, Martin Müller, Andrea B. Maier, Richard J. A. van Wezel, Jurgen A. H. R. Claassen, Jan Willem J. Elting

**Affiliations:** 1 Department of Human Movement Sciences, @AgeAmsterdam, Amsterdam Movement Sciences, Vrije Universiteit Amsterdam, Van der Boechorstraat 9, 1081 BT Amsterdam, The Netherlands; 2 Department of Biophysics, Donders Institute for Brain, Cognition and Behaviour, Radboud University, Heijendaalseweg 135, 6525 AJ Nijmegen, The Netherlands; 3 Department of Rehabilitation Medicine, Amsterdam UMC, Vrije Universiteit, Amsterdam Movement Sciences, P.O. Box 7057, 1007 MB Amsterdam, The Netherlands; 4 Department of Geriatric Medicine, Radboud University Medical Center, Reinier Postlaan 4, 6525 GC Nijmegen, The Netherlands; 5 Department of Neurology, Lucerne Kantonsspital, Spitalstrasse, CH-6000 Lucerne, Switzerland; 6 Department of Medicine and Aged Care, @AgeMelbourne, The Royal Melbourne Hospital, The University of Melbourne, City Campus, Level 6 North, 300 Grattan Street, Parkville, VIC 3050 Australia; 7 Department of Biomedical Signals and Systems, Technical Medical Centre, University of Twente, Zuidhorst Building, P.O. Box 217, 7500 AE Enschede, The Netherlands; 8 Department of Neurology, University Medical Center Groningen, Hanzeplein 1, 9713GZ Groningen, The Netherlands

**Keywords:** Cerebral autoregulation, Near-infrared spectroscopy, Transcranial Doppler, Cognitive dysfunction, Hypertension

## Abstract

**Purpose:**

Cerebral autoregulation (CA) aims to attenuate the effects of blood pressure variation on cerebral blood flow. This study assessed the criterion validity of CA derived from near-infrared spectroscopy (NIRS) as an alternative for Transcranial Doppler (TCD).

**Methods:**

Measurements of continuous blood pressure (BP), oxygenated hemoglobin (O_2_Hb) using NIRS and cerebral blood flow velocity (CBFV) using TCD (gold standard) were performed in 82 controls, 27 patients with hypertension and 94 cognitively impaired patients during supine rest (all individuals) and repeated sit to stand transitions (cognitively impaired patients). The BP-CBFV and BP-O_2_Hb transfer function phase shifts (TF_φ_) were computed as CA measures. Spearman correlations (*ρ*) and Bland Altman limits of agreement (BAloa) between NIRS- and TCD-derived CA measures were computed. BAloa separation < 50° was considered a high absolute agreement.

**Results:**

NIRS- and TCD-derived CA estimates were significantly correlated during supine rest (*ρ* = 0.22–0.30, *N* = 111–120) and repeated sit-to-stand transitions (*ρ* = 0.46–0.61, *N* = 19–32). BAloa separation ranged between 87° and 112° (supine rest) and 65°–77° (repeated sit to stand transitions).

**Conclusion:**

Criterion validity of NIRS-derived CA measures allows for comparison between groups but was insufficient for clinical application in individuals.

**Supplementary Information:**

The online version contains supplementary material available at 10.1007/s00421-021-04681-w.

## Introduction

Cerebral autoregulation (CA) is the mechanism aiming to keep cerebral blood flow constant during blood pressure (BP) fluctuations by constricting or dilating cerebral arterioles in response to BP increases and decreases, respectively (Bayliss 1902; Claassen et al. 2016; Moerman and De Hert 2019). CA acts complementary to the process of neurovascular coupling which aims to increase local cerebral blood flow in response to increased neural demand and is dependent on systemic and cerebral CO_2_ concentration (Rosengarten et al. 2001; Meng and Gelb 2015). Static and dynamic CA are distinguished to express the ability of CA to maintain cerebral blood flow during a changed steady-state BP and fluctuating BP, respectively (Tiecks et al. 1995; de Jong et al. 2017). Dynamic CA function is dependent on the frequency and speed of BP changes and can in contrast to static CA not be measured using MRI, PET or SPECT (Tiecks et al. 1995; de Jong et al. 2017). Impaired dynamic CA was reported in patients with stroke and traumatic brain injury (Radolovich et al. 2011; Castro et al. 2018) and may be a risk factor for mild cognitive impairment (MCI) and dementia, particularly in patients with hypertension and orthostatic hypotension (Freeman et al. 2011; Tarumi et al. 2014; Müller et al. 2020). Presently, dynamic CA is usually assessed using Transcranial Doppler (TCD) measurements, which provide a valid approximation of cerebral blood flow measured using MRI (Khan et al. 2017). However, TCD measurements require skilled investigators and are not feasible in a substantial proportion of older adults due to age-related temporal bone remodeling, limiting TCD availability and applicability in clinical populations (Claassen et al. 2016; Couture et al. 2017).

Near infrared spectroscopy (NIRS) is a potential alternative for TCD and measures changes in cerebral oxygenated and deoxygenated hemoglobin concentrations by detecting the intensity of reflected infrared light emitted into the brain. NIRS measurements were suggested to be potentially useful to assess CA in healthy young individuals (Kainerstorfer et al. 2015; Elting et al. 2018; Kim et al. 2018), healthy older adults (Gao et al. 2015), and various clinical populations (Zweifel et al. 2010; Rivera-Lara et al. 2017; Montgomery et al. 2020). NIRS-derived CA estimation may be performed using the cerebral oximetry index, or transfer function analysis (TFA) phase shift correcting for effects arising from the cerebral microcirculation using the information in the high-frequency range (0.2–0.5 Hz), in which CA is not active (Brady et al. 2010; Elting et al. 2018). However, there is very limited evidence on the validity of NIRS-derived CA estimation in an older, clinical population, for example with chronic diseases such as hypertension and cognitive impairment.

In this study, we assessed the criterion validity of NIRS-derived CA estimation in younger and older controls, patients with controlled hypertension, mild cognitive impairment (MCI) and Alzheimer’s dementia (AD) during supine rest and repeated sit to stand transitions. We hypothesize that NIRS-derived CA estimates correlate with TCD-derived CA measures, and have a high absolute agreement, i.e., a separation between upper and lower 95% limits of agreement < 50° (Sanders et al. 2019).

## Material and methods

### Study cohorts

BP, TCD and NIRS data from six cohorts, collected between 2008 and 2018 at three different centers, were included in this study: two cohorts of younger adults (younger controls; *n* = 39 and 14; mean age < 65 years), a cohort of older adults (older controls; *n* = 28; mean age > 65 years), a cohort of patients with controlled hypertension (*n* = 27), and cohorts of patients with MCI and AD (cognitively impaired patients; *n* = 37 and 57). The centers were (1) the Department of Neurology, Lucerne Kantonsspital, Lucerne, Switzerland (younger controls cohort 1 and patients with controlled hypertension); (2) the University Groningen Medical Center, Groningen, the Netherlands (younger controls cohort 2); (3) the Radboud University Medical Center, Nijmegen, the Netherlands (older controls and patients with MCI and AD). Table 1 lists the inclusion and exclusion criteria per cohort.

**Table 1:** Cohort characteristics

	Younger controls (cohort 1)	Younger controls (cohort 2)	Older controls	Patients with controlled hypertension	MCI patients	AD patients
Inclusion						
*N*	39	14	28	27	37	57
Measurement site	Luzerner Kantonsspital^a^	UMCG^b^	Radboudumc^c^	Lucerne Kantonsspital^a^	Radboudumc^c^	Radboudumc^c^
Inclusion/exclusion criteria	No smoking	Age between 20 and 50 years	Age > 50 years	Patients referred for diagnosis of cerebrovascular diseases	Age > 50 years	Age > 50 years
	Absence of any medical conditions	Absence of any medical conditions	No medical history of cardiovascular or cerebrovascular disease	History of SBP > 140 and/or DBP > 90 for > 2 years, successfully treated	Clinical diagnosis of MCI due to AD according to the NIA-AA criteria	Clinical diagnosis of AD according to the NIA-AA criteria
			Not using cardiovascular or psychotropic medication	No more than 50% stenosis of large arteries on duplex US	MOCA score 18–26	MMSE score between 12 and 26
				No smoking		
				No cardiac arrhythmias or heart failure		
Data collection						
BP device	Finometer Pro^d^	Portapres^d^	Finometer Pro^d^	Finometer Pro^d^	Finometer Pro^d^	Finometer Pro^d^
TCD device (sampling frequency)	Multidop^e^ (2 MHz)	Delica^f^ (2 MHz)	Multidop^e^ (2 MHz)	Multidop^e^ (2 MHz)	Doppler-BoxX^e^ (2 MHz)	Multidop^e^ (2 MHz)
NIRS device (sampling frequency)	NIRO-200NX^g^ (5 Hz)	Portalite^h^ (50 Hz)	Oxymon Mk III^g^ (10 Hz)	NIRO-200NX^g^ (5 Hz)	Oxymon Mk III^h^ (10 Hz)	Oxymon Mk III^h^ (10 Hz)
NIRS wavelengths	735, 810 and 850 nm	760 and 850 nm	765, 857 and 859 nm	735, 810 and 850 nm	765, 857 and 859 nm	765, 857 and 859 nm
NIRS inter optode distance	4 cm	4.0 cm	5 cm	4 cm	5 cm	5 cm

*MCI* mild cognitive impairment, *AD* Alzheimer’s dementia, *BP* blood pressure, *TCD* Transcranial Doppler, *NIRS* near-infrared spectroscopy, *SBP* systolic blood pressure, *DBP* diastolic blood pressure, *US* ultrasound, *MOCA* Montreal Cognitive Assessment, *MMSE* Mini-Mental State Examination

^a^Department of Neurology, Lucerne Kantonsspital, Lucerne, Switzerland

^b^University Medical Center Groningen, Groningen, the Netherlands

^c^Radboud University Medical Center, Nijmegen, the Netherlands

^d^Finapres Medical Systems, Amsterdam, The Netherlands

^e^Compumedics DWL, Singen, Germany

^f^Shenzhen, China

^g^Hasamotu Photonics, Herrsching, Germany

^h^Artinis Medical Systems, Elst, The Netherlands

In the quantitative analysis, data from the two cohorts of younger controls and patients with MCI and AD were pooled, leaving four pooled cohorts for statistical analysis: younger controls, older controls, hypertension patients and cognitively impaired patients.

All patients and controls signed informed consent and for all studies medical ethical approval was obtained and they were performed in accordance with the declaration of Helsinki.

### Participant characteristics

Information about age, sex, smoking habits, medical history and use of medication were obtained. Body mass index (BMI) and cognitive performance (MMSE and/or MOCA) were measured in older controls and patients with MCI and AD.

### Instrumentation

Blood pressure (BP), near-infrared spectroscopy (NIRS) and transcranial Doppler (TCD) were simultaneously measured. The used BP, TCD and NIRS devices and manufacturers per cohort are listed in Table 1 as well as the used sampling frequencies, wavelengths and inter optode distances.

Continuous, beat-to-beat BP was measured non-invasively using finger photoplethysmography. Near-infrared spectroscopy (NIRS) measurements were obtained bilaterally on the forehead to assess changes in cerebral oxygenated hemoglobin concentrations (O_2_Hb). The differential pathway factor (DPF), which accounts for the increased distance traveled by light due to scattering, is age-dependent and was computed using the following formula in adults aged below 50 years (Scholkmann and Wolf 2013): 4.99 + 0.067 × Age^0.814^. In other individuals, DPF was set to 6.61, the value for an age of 50 years, the highest age for which the formula is validated. Transcranial Doppler (TCD) measurements were performed bilaterally over the temporal bone to measure cerebral blood flow velocity (CBFV) in the middle cerebral arteries. The TCD probes were fixed using a head holder.

### Protocol

Room temperatures between 20 and 23 °C were pursued. Patients and controls were discouraged from talking and moving during the measurements.

Patients were asked to lie supine for at least 5 min during which blood pressure, cerebral blood flow velocity and oxygenated and deoxygenated cerebral hemoglobin (O_2_Hb and HHb, respectively) were measured.

Measurements during repeated sit-to-stand transitions were performed in patients with MCI and AD. The seat was adjusted to the patient’s height. Patients were asked to switch from sitting and standing position every 10 s (full cycle period of 20 s; frequency of 0.05 Hz) during 5 min to induce BP, CBFV and O_2_Hb oscillations.

### Data analysis

All analysis was performed using MATLAB (version R2019b, The MathWorks Inc., Natick, Massachusetts, USA).

#### Signal preprocessing

All signals were resampled to a uniform sampling frequency of 200 Hz and a moving median filter with a 0.15 s window was applied to remove spike artefacts from the signals.

#### Signal quality assessment

The quality of BP, CBFV and O_2_Hb signals was visually assessed by two authors (AM and JWE). This was performed separately for each signal, individual, side (left/right) and test condition. A signal was considered poor quality and discarded if it contained spike or step artefacts, or a recurrent heartbeat was not visible.

#### Transfer function analysis (TFA)

BP-CBFV (TCD-derived) and BP-O_2_Hb (NIRS-derived) transfer functions (TF) were computed for both sides in each individual using software from the CARNet community (Claassen et al. 2016). Signals were filtered using a 6th order Butterworth lowpass filter with a cutoff of 0.5 Hz and the signal mean was subtracted before performing the TFA. TF gain (TF_g_), phase shift (TF_φ_) and coherence (TF_c_) were computed as a function of frequency. TF_g_ and TF_φ_ with insignificant coherence in a frequency bin (tested according to the CARNet recommendations) (Claassen et al. 2016) were discarded from further analysis.

TF_φ_s were averaged over the sides with available data per individual and subsequently averaged over individuals to obtain grand average TF_φ_s per cohort. Averaging was performed using the circular mean (i.e., transforming angular quantities to positions on the unit circle, averaging x and y coordinates of these positions separately, and transforming the averaged x and y coordinates back to an angular quantity). NIRS and TCD-derived grand average TF_φ_s were plotted together to enable a visual assessment of their differences.

The very low frequency (VLF), low frequency (LF) and high frequency (HF) ranges were defined as 0.02–0.07 Hz, 0.07–0.2 Hz and 0.2–0.5 Hz, respectively (Elting et al. 2018). Mean TF_φ_ in the VLF and LF ranges were used as CA measures.

#### Correction for cerebral microcirculation effects

**Fig. 1:**
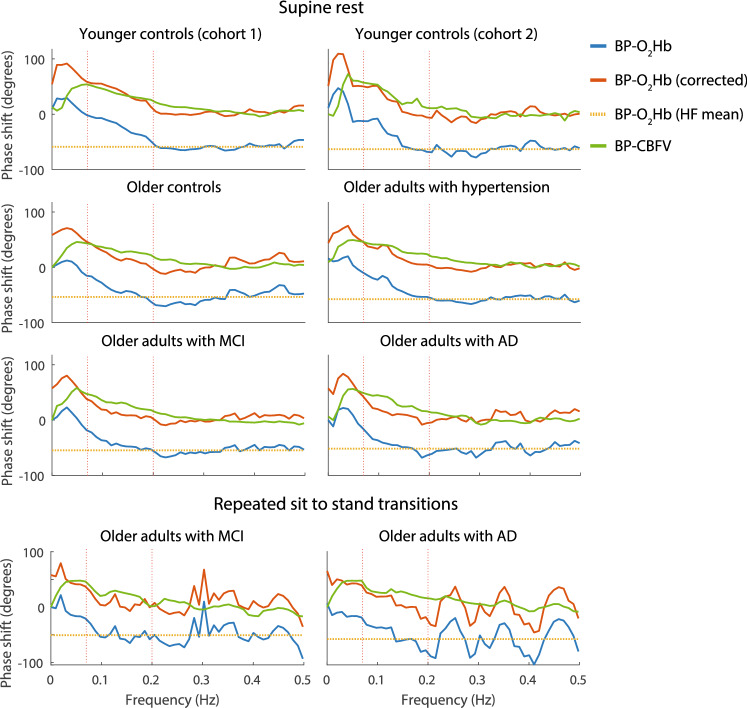
Grand average of BP-CBFV and BP-O_2_Hb TF_φ_ in supine rest and during repeated sit to stand transitions, per cohort. The blue and red traces are the BP-O_2_Hb TF_φ_s before and after correction, respectively. The yellow dotted lines are the means lines of the BP-O_2_Hb TF_φ_ in the high frequency (HF) range. *MCI* mild cognitive impairment, *AD* Alzheimer’s dementia

The BP-O_2_Hb TF_φ_s were corrected for cerebral microcirculation effects on waveform morphology: CBFV waveforms typically have a steeper upstroke compared to O_2_Hb waveforms, resulting in a relatively constant negative phase shift across frequencies (demonstrated in Supplementary File S1). Correction was performed by computing the mean phase shift in the HF range (in which CA is not active) and subtracting this mean phase shift from the TF_φ_ (Fig. 1). The HF range mean phase shift was determined based on the pooled cohort average TF_φ_.

#### S*tatistical analysis*

Circular means and standard deviations were used to aggregate phase shift data.

TF_φ_s were consecutively averaged over sides (left/right) and over frequencies within frequency ranges (VLF and LF), resulting in mean VLF and LF TF_φ_s for each individual.

Spearman rank correlations of NIRS- and TCD-derived CA estimates were computed as a measure of criterion validity. Furthermore, the absolute difference between NIRS- and TCD derived CA estimates were visualized using Bland Altman plots and 95% upper and lower limits of agreement were computed. A separation between upper and lower limits of agreement < 50 was considered high absolute agreement (Sanders et al. 2019). This value corresponds to the separation between 95% limits of agreement of Bland Altman analysis reflecting the test–retest reliability of TCD-derived CA measures (Sanders et al. 2019).

*p*-values < 0.05 were considered statistically significant.

## Results

### Participant characteristics

Table 2 lists the participant characteristics per cohort. Mean age in the two cohorts of younger controls was 48 years (SD 17.7) and 28 years (range 21–45), respectively, and ranged between 69.2 and 73.3 years in the cohorts of older controls and patients. Mean systolic/diastolic blood pressure in patients with controlled hypertension was 124.0/78.5 mmHg (SD 15.6/12.9). Median MOCA score was 23 points (interquartile range (IQR) 20.5–25.0) in MCI patients; median MMSE score was 16 points (IQR 14–18) in patients with AD, and 29 points (IQR 28–30) in older controls. Cerebral blood flow velocity as measured with TCD ranged between 38.2 cm/s in patients with AD to 62.8 cm/s in younger controls.

**Table 2:** Characteristics of patients and controls

Characteristic	Younger controls (cohort 1; *N* = 39)		Younger controls (cohort 2; *N* = 14)		Older controls (*N* = 28)		Patients with controlled hypertension (*N* = 27)		MCI patients (*N* = 37)		AD patients (*N* = 57)	
Age, years, mean (SD/range)	39	48.0 (17.7)	14	28 (21–45)	28	70.0 (3.7)	27	67.0 (14.7)	37	69.2 (8.4)	57	73.3 (6.1)
Female, *n* (%)	39	17 (43.6)	14	11 (78.6)	28	12 (42.9)	27	5 (18.5)	37	12 (32.4)	57	32 (56.1)
BMI, kg/m^2^, mean (SD)	39	< 30		NA	28	26.2 (2.9)	27	< 30	37	26.0 (3.7)	57	24.8 (3.7)
Current smoking, *n* (%)	39	0 (0)		NA		NA	27	0 (0)	31	4 (12.9)		NA
Cardiovascular or cerebrovascular disease, *n* (%)	39	0 (0)	14	0 (0)	28	0 (0)	27	27 (100)	30	9 (30.0)	57	43 (75.4)
Cardiovascular or psychotropic medication, *n* (%)	39	0 (0)	14	0 (0)	28	0 (0)	27	27 (100)	19	13 (68.4)	57	16 (28.1)
MMSE, points, median [IQR]		NA		NA	28	29 [28–30]		NA		NA	57	16 [14–18]
MOCA, points, median [IQR]		NA		NA		NA		NA	37	23 [20.5–25]		NA
Blood pressure and cerebral blood flow velocity												
SBP, mmHg, mean (SD)	39	113.8 (16.6)^a^	14	129.8 (21.8)^a^	28	132.9 (12.8)^b^	26	124.0 (15.6)^a^	37	142.8 (22.0)^b^	57	138 (13.1)^b^
DBP, mmHg, mean (SD)	39	68.4 (12.1)^a^	14	79.4 (18.2)^a^	28	78.5 (9.6)^b^	26	67.5 (12.9)^a^	37	83.8 (12.2)^b^	57	78.4 (6.4)^b^
Cerebral blood flow velocity, cm/s, mean (SD)	39	62.8 (13.6)	14	62.3 (8.7)	23	46.0 (8.8)	26	53.2 (12.8)	33	41.8 (12.3)	39	38.2 (9.6)

The table lists patient characteristics for each of the included cohorts. *SD* standard deviation, *IQR* interquartile range, *BMI* Body Mass Index, *MMSE* mini-mental state examination, *MOCA* Montreal cognitive assessment, *HR* heart rate, *bpm* beats per minute, *SBP* systolic blood pressure, *DBP* diastolic blood pressure, *CBFV* cerebral blood flow velocity, *VLF* very low-frequency range, *LF* low-frequency range, *HF* high-frequency range, *MCI* mild cognitive impairment, *AD* Alzheimer’s dementia, *BP* blood pressure

^a^Measured using continuous BP monitor

^b^Measured using a sphygmomanometer

### Signal quality

During supine rest, each signal (BP, CBFV, O_2_Hb) was available in good quality in 148/202 participants (73.2%; younger controls: 26/41; older controls 19/28; hypertension patients: 23/27; cognitively impaired patients: 59/94). During the repeated sit-to-stand transitions, each signal was available in good quality in 35/94 cognitively impaired patients (37.2%).

### Correction for cerebral microcirculation effects

Figure 1 shows the results of correcting for cerebral microcirculation effects on grand average level. After correction, the grand average BP-O_2_Hb TF_φ_ approximated the grand average BP-CBFV TF_φ_: − 43°/− 71° error before correction to 16°/− 12° error after correction (VLF/LF range, all participants, supine rest) and -62/-75 degrees error before correction to − 4°/− 17° error after correction (VLF/LF range, cognitively impaired patients, sit to stand transitions). Errors before and after correction per cohort and experimental condition are listed in Table 3.

**Table 3:** Cerebral autoregulation estimates derived from TCD and NIRS

	Supine rest										Sit to stand transitions	
	Younger controls (*N* = 53)		Older controls (*N* = 28)		Patients with controlled hypertension (*N* = 27)		Cognitively impaired patients (*N* = 94)		All (*N* = 202)		Cognitively impaired patients (*N* = 94)	
BP-CBFV and BP-O_2_Hb												
VLF												
GA error bc, mean (SD), degrees^a^	39^b^	− 40 (20)	17^b^	− 42 (13)	18^b^	− 41 (12)	46^b^	− 46 (17)	120^b^	− 43 (16)	32^b^	− 62 (5)
GA error ac, mean (SD), degrees^a^	39^b^	23 (20)	17^b^	18 (12)	18^b^	14 (11)	46^b^	12 (17)	120^b^	16 (16)	32^b^	− 4 (5)
Spearman correlation	39	0.00	17	0.24	18	0.55*	46	0.24	120	0.22*	32	0.46**
BA bias (loa), degrees	39	24 (149)	17	16 (65)	18	13 (82)	46	7 (91)	120	14 (112)	32	− 0 (78)
LF												
GA error bc, mean (SD), degrees^a^	36^b^	− 65 (7)	15^b^	− 72 (7)	17^b^	− 69 (8)	46^b^	− 76 (13)	111^b^	− 71 (4)	19^b^	− 75 (7)
GA error ac, mean (SD), degrees^a^	36^b^	− 2 (7)	15^b^	− 12 (6)	17^b^	− 12 (7)	46^b^	− 19 (13)	111^b^	− 12 (3)	19^b^	− 17 (7)
Spearman correlation	36	0.37*	15	0.16	17	− 0.06	43	0.30	111	0.30**	19	0.62**
BA bias (loa), degrees	36	2 (109)	15	− 10 (52)	17	− 2 (73)	43	− 11 (71)	111	− 5 (86)	19	− 4 (65)

Grand average (GA) errors before and after correction (bc and ac, respectively), and Spearman correlations and Bland Altman (BA) analysis results between NIRS- and TCD-derived CA measures within pooled cohorts. *BP* blood pressure, *CBFV* cerebral blood flow velocity, *O*_*2*_*Hb* oxygenated hemoglobin. *loa* separation between 95% upper and lower limits of agreement. One and two stars indicate statistically significant correlations with p values lower than 0.05 and 0.01, respectively

^a^Mean and standard deviation over frequencies within the frequency range

^b^Represents the number of patients for whom both BP-CBFV and BP-O_2_Hb TFs was available. The number of patients for whom the separate TFs were available (enabling them to be included in the TF grand average) was higher (see Supplementary Table S2) ed cohort *SD* standard deviation

### Correlation between NIRS- and TCD- derived CA estimates

Spearman correlations between NIRS-and TCD derived CA estimates during supine rest were significant in patients with hypertension in the VLF range (*ρ* = 0.55, *n* = 27) and in younger controls in the LF range (*ρ* = 0.37, *n* = 53), but not in the other cohorts. In the pooled group of all patients and controls, Spearman correlations during supine rest were 0.22 (VLF, *n* = 120, *p* = 0.016) and 0.30 (LF, *n* = 111, *p* = 0.002). During repeated sit-to-stand transitions in cognitively impaired patients, Spearman correlations were 0.46 (VLF, *n* = 32, *p* = 0.009) and 0.61 (LF, *n* = 19, *p* < 0.001), respectively. Table 3 lists the correlations between NIRS- and TCD-derived CA estimates in the entire population as well as in the different pooled cohorts. Supplementary Table S2 lists the mean TF_g,_ TF_φ_ and TF_c_ for each pooled cohort, test condition, TF, and frequency range. Supplementary Table S3 lists the Spearman correlation stratified for the NIRS measurement device. NIRS- and TCD-derived CA measures only were significantly correlated when measured with the Oxymon Mk III device (VLF/LF, *n* = 63/58, *ρ* = 0.22/0.30).

### Bland Altman analysis

**Fig. 2:**
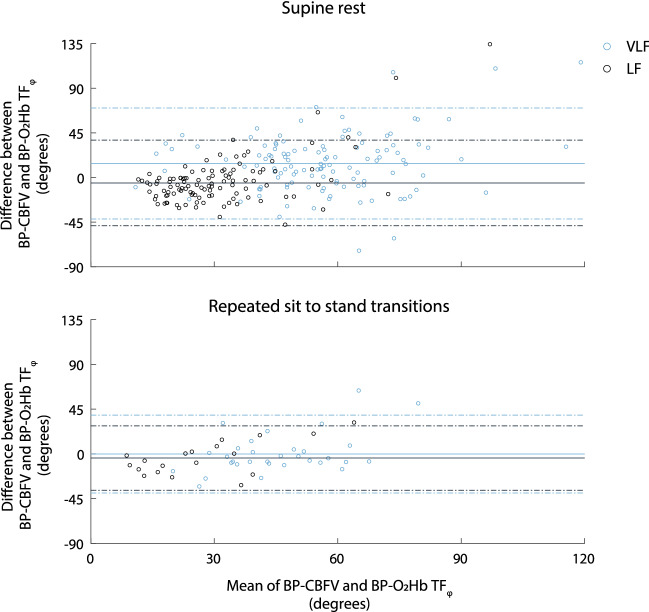
Bland Altman plots showing agreement between NIRS- and TCD- derived CA measures during supine rest and repeated sit-to-stand transitions. The horizontal solid lines indicate the mean difference; the horizontal dashed lines indicate the 95% limits of agreement. *VLF* very low-frequency range, *LF* low-frequency range

Bland Altman plots of the differences between NIRS- and TCD-derived CA estimates within the entire population of all patients and controls are shown in Fig. 2. For measurements in supine rest (pooled group of all patients and controls), the 95% limits of agreement were − 42° to 70° (VLF) and − 49 to 38° (LF). For measurements during repeated sit-to-stand transitions (cognitively impaired patients), the 95% limits of agreement were − 39 to 38° (VLF) and − 37 to 28° (LF). The 95% limits of agreement per cohort and experimental condition are listed in Table 3.

## Discussion

This study assessed the criterion validity of near-infrared spectroscopy (NIRS) as a cerebral autoregulation (CA) estimation method. Correction of NIRS-derived CA estimates for waveform morphology differences between CBFV and O_2_Hb signals reduced the errors in the grand average. Significant but low correlations (0.22–0.30) between NIRS and TCD-derived CA measures were found for measurements performed during supine rest. During repeated sit-to-stand transitions, correlations were higher (0.46–0.61), but the analyzed number of individuals was low (19–32). Bland Altman analyses showed a low absolute agreement between NIRS- and TCD-derived CA measures (separation between upper and lower 95% limits of agreement ranging from 65° to 112°).

### Agreement between NIRS- and TCD derived CA measures

Correction of the BP-O_2_Hb TF_φ_ by subtracting the negative mean phase in the HF range reduced errors on grand average level both during supine rest and repeated sit-to-stand transitions with at least 27° and 58°, respectively. This negative mean phase was shown to reflect waveform morphology differences between the systemic and cerebral circulations. The significant correlations between NIRS- and TCD-derived CA measures suggest that NIRS-derived CA estimates may be used to compare groups. Absolute errors between NIRS and TCD-derived CA measures in individuals were higher than the used cutoff criterion for clinical application in individuals (Sanders et al. 2019). This finding was different from previous work that investigated the transfer function from O_2_Hb to deoxygenated haemoglobin (HHb) (Elting et al. 2018). In that study, a relatively good agreement between NIRS based autoregulation estimates and BP-CBFV based autoregulation estimates was found, with intra class correlation values up to 0.7. Apart from the difference in signals that were analysed, the most likely explanation for these different results is a difference in signal quality and artefact levels between these studies. In the current dataset, artefact levels were relatively high, with HHb signals that were largely of insufficient quality, which prevented us from examining O_2_Hb-HHb transfer functions.

The Bland–Altman plots showed higher errors when phase differences were higher, indicating a poor agreement between NIRS- and TCD-derived CA estimates on the individual level. This poor agreement is likely to arise from a combination of factors, i.e., noise in the NIRS signals, differences between NIRS devices used, effects of extra-cranial circulation, differences in the part of the brain monitored between NIRS and TCD, and the use of TCD as the gold standard because of the lack of the optimal gold standard. These factors are discussed in more detail below.

### Supine rest versus sit to stand transitions

The correlation between NIRS- and TCD- derived CA measures was particularly large when measured during repeated sit-to-stand transitions. The transitions increased the coherence between BP, CBFV and O_2_Hb in the VLF range, implying a better validity of the TFA linearity assumption, as reported before (Claassen et al. 2016). The relatively high correlation between NIRS- and TCD-derived CA measures during repeated sit-to-stand transitions may also be explained by the higher BP variability, which was reported to be a positive determinant of CA reliability in a previous study (Elting et al. 2020). CA impairment may become manifest during transitions rather than in supine rest, indicating the potential clinical relevance of assessing CA during transitions (Mol et al. 2018, 2019, 2020; de Heus et al. 2020). In contrast to supine rest, transitions may induce rapid BP drops, challenging CA beyond its capacity due to its intrinsic latency (Kuo et al. 2003). However, the number of individuals with good quality signals during sit-to-stand transitions in the present study was low.

### VLF versus LF range

The correlation between NIRS- and TCD-derived CA measures was higher in the LF range compared to the VLF range. Non-linear behavior in the VLF range may play a role as reported in a previous study and indicated by the low coherence in the VLF range compared to the LF and HF range (Giller and Mueller 2003). As demonstrated in supplementary file S1, estimation of the CBFV-O_2_Hb TF_φ_ using the HF mean of the BP-O_2_Hb TF_φ_ was less accurate in the VLF compared to the LF range. The findings may also be explained by the relatively short duration of the measurements relative to the oscillation period in the VLF range, implying a potentially large effect of artefacts on the BP-O_2_Hb TF_φ_ in this frequency range. Further studies should preferably prolong the measurements, enabling the selection of data segments with more BP variation, improving the reproducibility of CA assessment (Elting et al. 2020).

### Difference between cohorts

The correlation between NIRS- and TCD- derived CA measures was significant in the entire population of patients and controls during supine rest, but not in each of the pooled cohorts. Apart from low sample sizes, device and data quality differences between the different centers may have played a role. For example, differences in NIRS inter optode distance (i.e., the distance between transmitter and receiver), which partly determines the volume of brain tissue being sampled, may have influenced the results (Klaessens et al. 2003). The analysis stratified for NIRS device supports the idea that the device type and inter optode distance may play a role as the correlation between NIRS- and TCD-derived CA measures was only significant when measured with one type of device (Oxymon MK III), which in contrast to the other NIRS devices used had an inter optode distance of 5 cm. However, this difference in significance of correlations may alternatively well be attributed to the difference in a number of individuals measured, which was highest in Oxymon MK III group as absolute correlation values were comparable between devices.

### NIRS measurements for CA estimation

Though NIRS aims to measure cerebral oxygenation changes only, the influence of changes in the extra-cranial circulation on the NIRS signal cannot be entirely excluded, posing a potential limitation to the study. Extra-cranial effects were demonstrated in a previous study, which showed an effect of blocking the extra-cranial circulation using a head cuff on the measured NIRS signals (Davie and Grocott 2012). However, these extra-cranial effects were reported to be relatively small when CA is assessed in the frequency domain using an inter optode distance > 3 cm (Obrig et al. 2000). Remaining extra-cranial effects may have contributed to the poor correlation between NIRS- and TCD-derived CA measures found in the present study. As the extra-cranial circulation is not influenced by CA, extra-cranial circulation is synchronous to the systemic circulation (i.e., BP). Extra-cranial circulation effects on the NIRS measurements would therefore draw the BP-O_2_Hb TF_φ_ to zero in the VLF and LF ranges (Obrig et al. 2000). This is was not observed in the present study where the BP-O_2_Hb TF_φ_ quite closely approximated the BP-CBFV TF_φ_ (Fig. 1), suggesting only a minor effect of extra-cranial effects on the NIRS-derived CA measures. Extra-cranial effects should be aimed to be further excluded in future studies, which may in part be performed by using both small and large inter optode distances. Optodes with small distances primarily measure extra-cranial effects. These effects can then be subtracted from measurements from larger distance optodes, which are assumed to measure both cerebral and extra-cranial effects.

In the present study, NIRS measurements were performed bilaterally on the forehead to measure frontal cortical cerebral oxygenation changes as an indication of general cerebral perfusion. However, there may be differences between middle and anterior cerebral artery supplied brain regions (Wolf et al. 2002; Obrig et al. 2002), potentially explaining part of the differences between NIRS- and TCD-derived CA measures. Furthermore, the cortical neurovascular coupling may give rise to small differences between local cortical perfusion measured using NIRS and regional cerebral perfusion measured using TCD (Anderson et al. 1987; Iadecola 2017).

### Signal quality

A considerable proportion of the included individuals did not have an adequate quality of all signals (BP, CBFV, O_2_Hb) during supine rest, which is a limitation of this study. The proportion was lowest (62.7%) in cognitively impaired patients, which might be explained by poor understanding of instructions not to move or a lower baseline cerebral blood flow causing a lower signal to noise ratio in these individuals. The even lower availability of all signals (37.2%) during repeated sit-to-stand transitions in this pooled cohort can be attributed to the abundant occurrence of transition-induced movement artefacts. Due to the removal of negative phase shifts, the number of individuals for whom both NIRS- and TCD-derived CA measures were available was further reduced, posing a substantial limitation to the applicability of NIRS-based CA estimation. Further efforts should be made to decrease NIRS sensitivity to movement artefacts to enhance its applicability for CA estimation.

### TCD as gold standard

TCD is the gold standard for CA assessment, but also has limitations. TCD-derived CA measures have limited reproducibility (Sanders et al. 2018, 2019; Elting et al. 2020), potentially indicating that physiological factors apart from CA may influence cerebral blood flow velocity and hence TCD-derived CA measures. These factors may also partly explain the poor correlation with NIRS-derived CA estimates. TCD signals had to be discarded due to artefacts in some individuals with good quality BP and NIRS signals, limiting the number of individuals that could be included in the comparative analyses.

### Strength and limitations

The strength of this study is the diversity of the included cohorts and the simultaneous measurements of BP, TCD and NIRS both during well standardized (supine rest) and CA challenging (repeated sit-to-stand transitions) test conditions. Limitations include the relatively short duration of the measurements, the differences between the NIRS devices used in the different cohorts, the susceptibility of NIRS devices to potentially measure extra-cranial along with cerebral oxygenation effects, and the relatively large proportion of data that could not be used in the final analysis due to the presence of artefacts.

### Conclusion and clinical implications

Criterion validity of NIRS-derived CA estimates increases after correction for non-CA effects arising from the cerebral microvasculature. Criterion validity may be sufficient to enable comparisons between groups, but this could not be fully established due to the diversity of the included cohorts as well as the differences between the NIRS devices used. Criterion validity was insufficient for clinical application in individuals. The results suggest that NIRS-derived CA estimates may be more valid during repeated sit-to-stand transitions. However, artefacts in NIRS recordings impede CA estimation as indicated by the substantial proportion of the data that had to be discarded. Reducing motion artefacts is needed to increase the quality of measurements during transitions and the applicability of NIRS-derived CA estimation. Increasing the quality of NIRS-derived CA estimation during supine rest could be performed by prolonging measurements and selecting data segments with more BP variation. After optimization of measurement duration, NIRS device settings, measurement protocol and artefact removal, NIRS measurements in geriatric outpatients may potentially enable valid CA assessment in a wider range of patients during more instances.

## Supplementary Information

Below is the link to the electronic supplementary material.

Supplementary file1 (DOCX 712 kb)

Supplementary file2 (DOCX 32 kb)

Supplementary file3 (DOCX 14 kb)

## Data Availability

Data will be made available upon reasonable request.

## Abbreviations

AD: Alzheimer’s dementia
BAloa: Limits of agreement of Bland Altman analysis
BMI: Body mass index
BP: Blood pressure
CA: Cerebral autoregulation
CBFV: Cerebral blood flow velocity
DPF: Differential pathway factor
HF: High frequency
HHb: Deoxygenated hemoglobin
LF: Low frequency
MCI: Mild cognitive impairment
MMSE: Mini-Mental State Examination
MOCA: Montreal Cognitive Assessment
NIRS: Near-infrared spectroscopy
O2Hb: Oxygenated hemoglobin
TCD: Transcranial Doppler
TF: Transfer function
TFA: Transfer function analysis
VLF: Very low frequency

## Code availability

Code will be made available upon reasonable request.

## References

[CR1] Anderson RE, Sundt TM, Yaksh TL (1987). Regional cerebral blood flow and focal cortical perfusion: a comparative study of 133 Xe, 85 Kr, and umbelliferone as diffusible indicators. J Cereb Blood Flow Metab.

[CR2] Bayliss WM (1902). On the local reactions of the arterial wall to changes of internal pressure. J Physiol.

[CR3] Brady K, Joshi B, Zweifel C (2010). Real-time continuous monitoring of cerebral blood flow autoregulation using near-infrared spectroscopy in patients undergoing cardiopulmonary bypass. Stroke.

[CR4] Castro P, Azevedo E, Sorond F (2018). Cerebral autoregulation in stroke. Curr Atheroscler Rep.

[CR5] Claassen JA, Meel-van den Abeelen AS, Simpson DM, Panerai RB (2016). Transfer function analysis of dynamic cerebral autoregulation: a white paper from the International Cerebral Autoregulation Research Network. J Cereb Blood Flow Metab.

[CR6] Couture EJ, Desjardins G, Denault AY (2017). Transcranial Doppler monitoring guided by cranial two-dimensional ultrasonography. Can J Anesth Can d’anesthésie.

[CR7] Davie SN, Grocott HP (2012). Impact of extracranial contamination on regional cerebral oxygen saturation. Anesthesiology.

[CR8] de Heus RAA, de Jong DLK, Rijpma A (2020). Orthostatic blood pressure recovery is associated with the rate of cognitive decline and mortality in clinical Alzheimer’s disease. J Gerontol Ser A.

[CR9] de Jong DLK, Tarumi T, Liu J (2017). Lack of linear correlation between dynamic and steady-state cerebral autoregulation. J Physiol.

[CR10] Elting JWJ, Tas J, Aries MJH (2018). Dynamic cerebral autoregulation estimates derived from near infrared spectroscopy and transcranial Doppler are similar after correction for transit time and blood flow and blood volume oscillations. J Cereb Blood Flow Metab.

[CR11] Elting JW, Sanders ML, Panerai RB (2020). Assessment of dynamic cerebral autoregulation in humans: Is reproducibility dependent on blood pressure variability?. PLoS ONE.

[CR12] Freeman R, Wieling W, Axelrod FB (2011). Consensus statement on the definition of orthostatic hypotension, neurally mediated syncope and the postural tachycardia syndrome. Clin Auton Res.

[CR13] Gao Y, Zhang M, Han Q (2015). Cerebral autoregulation in response to posture change in elderly subjects-assessment by wavelet phase coherence analysis of cerebral tissue oxyhemoglobin concentrations and arterial blood pressure signals. Behav Brain Res.

[CR14] Giller CA, Mueller M (2003). Linearity and non-linearity in cerebral hemodynamics. Med Eng Phys.

[CR15] Iadecola C (2017). The neurovascular unit coming of age: a journey through neurovascular coupling in health and disease. Neuron.

[CR16] Kainerstorfer JM, Sassaroli A, Tgavalekos KT, Fantini S (2015). Cerebral autoregulation in the microvasculature measured with near-infrared spectroscopy. J Cereb Blood Flow Metab.

[CR17] Khan MA, Liu J, Tarumi T (2017). Measurement of cerebral blood flow using phase contrast magnetic resonance imaging and duplex ultrasonography. J Cereb Blood Flow Metab.

[CR18] Kim JM, Choi JK, Choi M (2018). Assessment of cerebral autoregulation using continuous-wave near-infrared spectroscopy during squat-stand maneuvers in subjects with symptoms of orthostatic intolerance. Sci Rep.

[CR19] Klaessens JHGM, Thijssen JM, Hopman JCW, Liem KD (2003). Experimental verification of conditions for near infrared spectroscopy (NIRS). Technol Heal Care.

[CR20] Kuo TBJ, Chern C-M, Yang CCH (2003). Mechanisms underlying phase lag between systemic arterial blood pressure and cerebral blood flow velocity. Cerebrovasc Dis.

[CR21] Meng L, Gelb AW (2015). Regulation of cerebral autoregulation by carbon dioxide. Anesthesiology.

[CR22] Moerman A, De Hert S (2019). Why and how to assess cerebral autoregulation?. Best Pract Res Clin Anaesthesiol.

[CR23] Mol A, Reijnierse EM, Bui Hoang PTS (2018). Orthostatic hypotension and physical functioning in older adults: a systematic review and meta-analysis. Ageing Res Rev.

[CR24] Mol A, Woltering JHH, Colier WNJM (2019). Sensitivity and reliability of cerebral oxygenation responses to postural changes measured with near-infrared spectroscopy. Eur J Appl Physiol.

[CR25] Mol A, Slangen LRN, Trappenburg MC (2020). Blood pressure drop rate after standing up is associated with frailty and number of falls in geriatric outpatients. J Am Heart Assoc.

[CR26] Montgomery D, Brown C, Hogue CW (2020). Real-time intraoperative determination and reporting of cerebral autoregulation state using near-infrared spectroscopy. Anesth Analg.

[CR27] Müller M, Österreich M, Lakatos L, Von HA (2020). Cerebral macro- and microcirculatory blood flow dynamics in successfully treated chronic hypertensive patients with and without white mater lesions. Sci Rep.

[CR28] Obrig H, Neufang M, Wenzel R (2000). Spontaneous low frequency oscillations of cerebral hemodynamics and metabolism in human adults. Neuroimage.

[CR29] Obrig H, Israel H, Kohl-Bareis M (2002). Habituation of the visually evoked potential and its vascular response: implications for neurovascular coupling in the healthy adult. Neuroimage.

[CR30] Radolovich DK, Aries MJH, Castellani G (2011). Pulsatile intracranial pressure and cerebral autoregulation after traumatic brain injury. Neurocrit Care.

[CR31] Rivera-Lara L, Geocadin R, Zorrilla-Vaca A (2017). Validation of near-infrared spectroscopy for monitoring cerebral autoregulation in comatose patients. Neurocrit Care.

[CR32] Rosengarten B, Huwendiek O, Kaps M (2001). Neurovascular coupling and cerebral autoregulation can be described in terms of a control system. Ultrasound Med Biol.

[CR33] Sanders ML, Claassen JAHR, Aries M (2018). Reproducibility of dynamic cerebral autoregulation parameters: a multi-centre, multi-method study. Physiol Meas.

[CR34] Sanders ML, Elting JWJ, Panerai RB (2019). Dynamic cerebral autoregulation reproducibility is affected by physiological variability. Front Physiol.

[CR35] Scholkmann F, Wolf M (2013). General equation for the differential pathlength factor of the frontal human head depending on wavelength and age. J Biomed Opt.

[CR36] Tarumi T, Dunsky DI, Khan MA (2014). Dynamic cerebral autoregulation and tissue oxygenation in amnestic mild cognitive impairment. J Alzheimer’s Dis.

[CR37] Tiecks FP, Lam AM, Aaslid R, Newell DW (1995). Comparison of static and dynamic cerebral autoregulation measurements. Stroke.

[CR38] Wolf M, Wolf U, Toronov V (2002). Different time evolution of oxyhemoglobin and deoxyhemoglobin concentration changes in the visual and motor cortices during functional stimulation: a near-infrared spectroscopy study. Neuroimage.

[CR39] Zweifel C, Castellani G, Czosnyka M (2010). Continuous assessment of cerebral autoregulation with near-infrared spectroscopy in adults after subarachnoid hemorrhage. Stroke.

