# Supplementary material for: Cerebral autoregulation assessed by near-infrared spectroscopy: validation using transcranial Doppler in patients with controlled hypertension, cognitive impairment and controls
Source: Eur J Appl Physiol. 2021 Apr 16;121(8):2165–76. doi: 10.1007/s00421-021-04681-w (PMC8260523; doi:10.1007/s00421-021-04681-w)
Supplement: Supplementary file 1 — Supplementary file1 (DOCX 712 kb) [file 421_2021_4681_MOESM1_ESM.docx]

**Supplementary file S1**

Cerebral autoregulation (CA) may be estimated using the blood pressure (BP) to oxygenated hemoglobin (O_2_Hb) transfer function phase shift (TF_φ_). However, as O_2_Hb not only reflects cerebral blood flow, but is also affected by the cerebral microcirculation, the BP-O_2_Hb TF_φ_ should be corrected for cerebral microcirculation effects to approximate the BP to cerebral blood flow velocity (CBFV) TF_φ_ used as a gold standard measure of CA. The different TF_φ_ and their relationship with physiology are shown in figure S1.1.

Effects from the cerebral microcirculation are reflected by the CBFV-O_2_Hb TF_φ,_ which can be subtracted from the BP-O_2_Hb TF_φ_ (figure S1.2) As CBFV and O_2_Hb are similarly influenced by cerebral autoregulation, this subtraction does not result in a loss of cerebral autoregulation information reflected by the BP-O_2_Hb TF_φ_. However, computation of CBFV-O_2_Hb TF_φ_ requires transcranial Doppler (TCD) measurements, a requirement which was aimed to be eliminated. The CBFV-O_2_Hb TF_φ_ should therefore be estimated without the use of TCD measurements. As demonstated in Figure S1.2, the CBFV-O_2_Hb TF_φ_ can be estimated using the mean of the BP-O_2_Hb TF_φ_ in the high frequency range (HF, 0.2 – 0.5 Hz), which is typically negative. Correcting the BP-O_2_Hb TF_φ_ for cerebral microcirculation effects can hence be performed by subtracting its mean in the HF range.

To test the hypothesis that cerebral microcirculation effects reflected by the CBFV-O_2_Hb TF_φ_ particularly comprise waveform steepness decline between macro- and microcirculation, signals with different waveform steepness were simulated and their TF_φ_ was computed (Figure S1.3). The CBFV and O_2_Hb signals were simulated using 3000 heart beats with a random period between 0.8 and 1.2 seconds and superposed white noise. Heart beats in the CBFV and O_2_Hb signal were simulated using triangles with a peaks at 5% and 45% of the heart cycle, respectively.

Figure S1.3 demonstrates by simulation that a lower waveform steepness in the O_2_Hb signal (triangular waveform middle panel) compared to the CBFV signal (sawtooth waveform upper panel) accounts for the negative phase shift in CBFV-O_2_Hb transfer function in the high frequency range. This negative phase shift was also observed in the experimental data (yellow curves in Figure S1.2). This finding indicates that waveform steepness decline in the cerebral microcirculation is an important effect to be corrected for when estimating CA using the BP-O_2_Hb TF_φ._


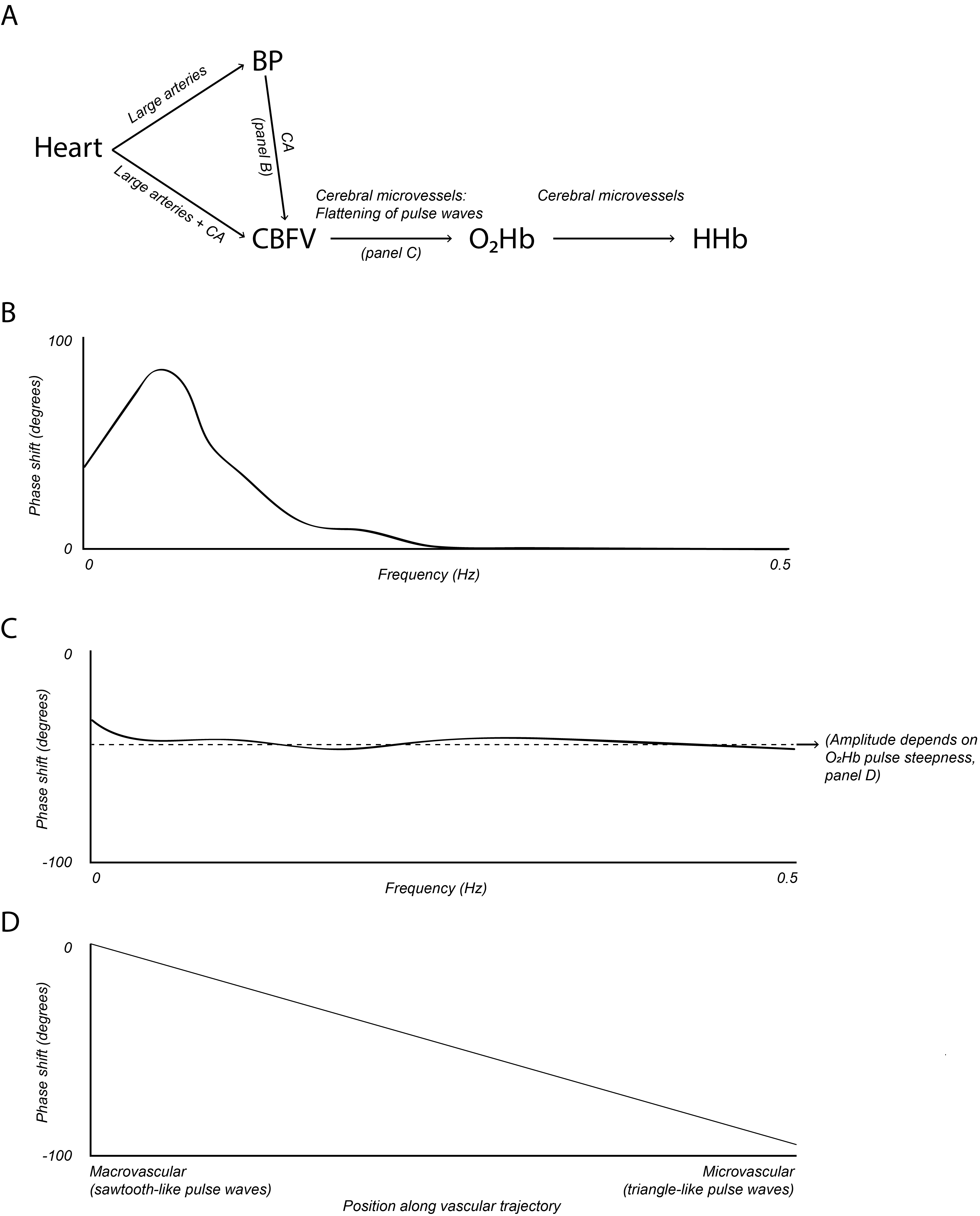


**Figure S1.1. Conceptual illustration of TF_φ_ in relation to physiology.** Panel A represents the different physiological signals that can be measured. The arrows denote the frequency-dependent delay between the signals as can be quantified using the TF_φ_. In serial signals, these TF_φ_s add up: the BP-CBFV TF_φ_ represents only CA induced phase shift; the CBFV-O_2_Hb TF_φ_ represents waveform induced phase shift; the BP-O_2_Hb TF_φ_ represents the sum of both. Panel B shows a schematic representation of the BP-CBFV TF_φ_, representing CA. Panel C shows a schematic representation of the CBFV-O_2_Hb TF_φ,_ which represents cerebral microcirculation effects (waveform changes) and can be approximated using a horizontal line. The amplitude of this horizontal line depends on the steepness of the O_2_Hb pulse waves, as schematically shown in panel D. BP: blood pressure; CBFV: cerebral blood flow velocity; O_2_Hb: oxygenated hemoglobin; HHb: deoxygenated hemoglobin


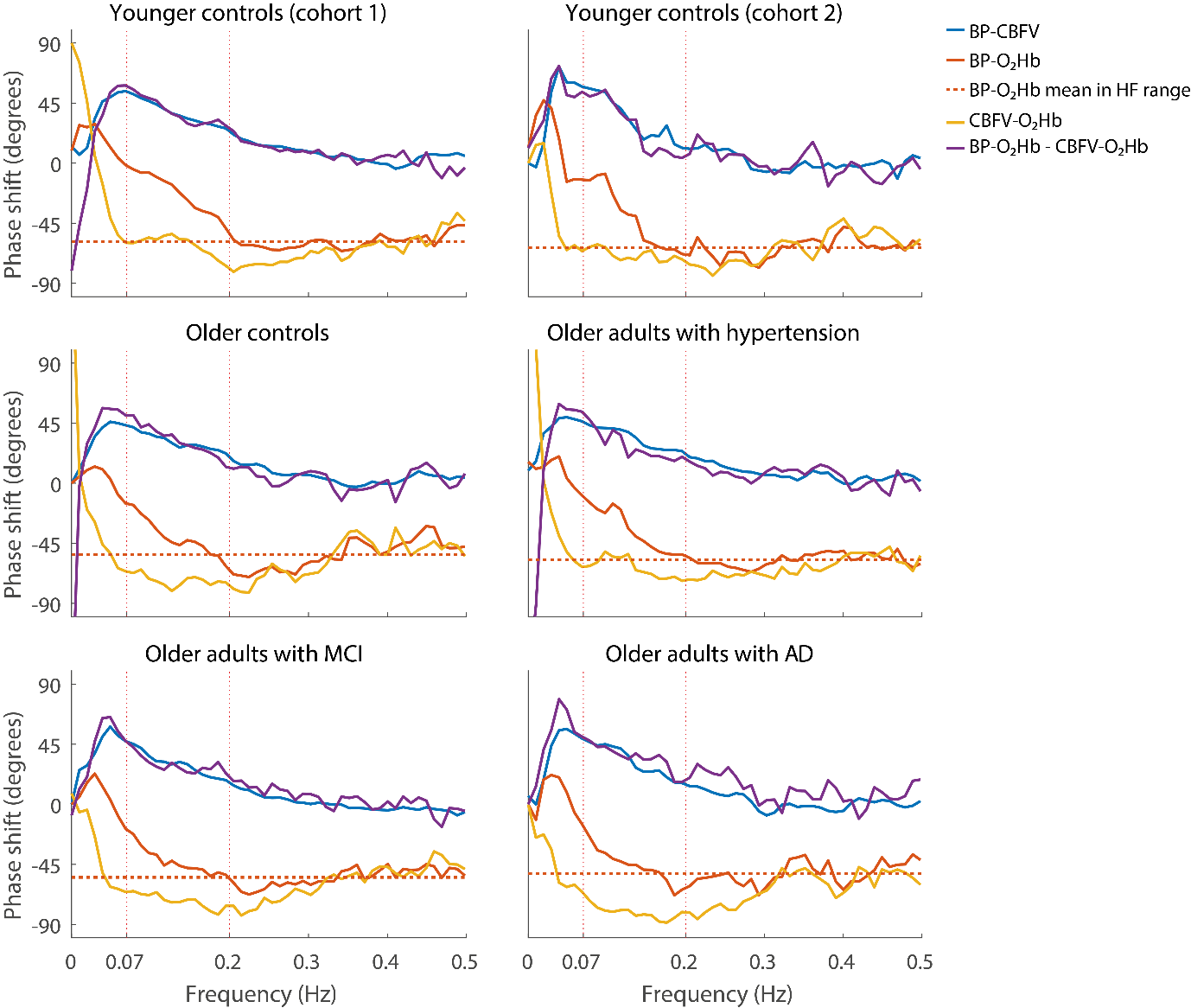


**Figure S1.2. Grand average of BP-CBFV, BP-O_2_Hb and CBFV-O_2_Hb TF_φ_ in supine rest, per cohort.** The red dotted lines are the means lines of the BP-O_2_Hb TF_φ_ in the high frequency (HF) range. MCI: mild cognitive impairment; AD: Alzheimer’s dementia.

**
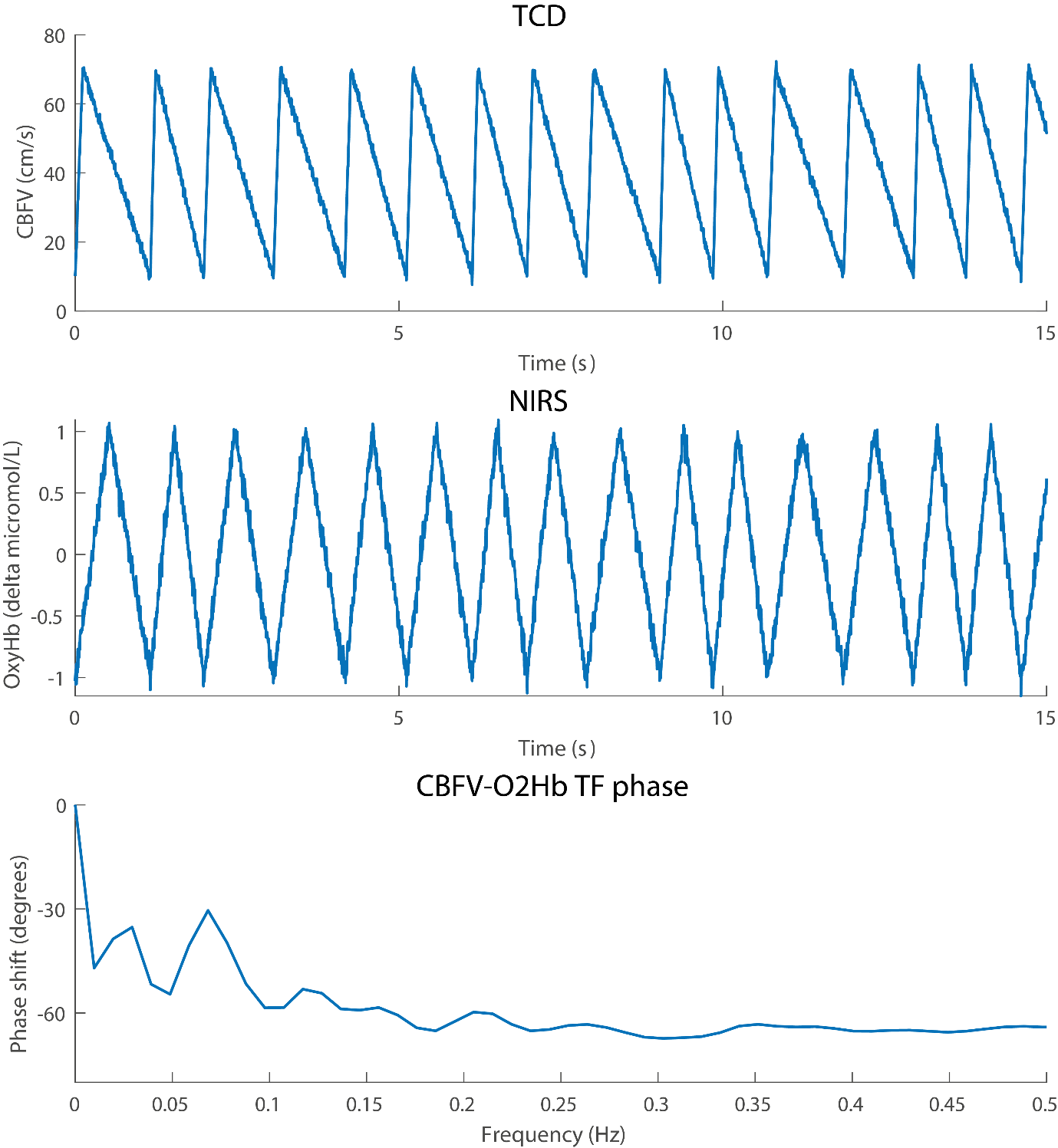
**

**Figure S1.3. Simulated CBFV and O_2_Hb signals and their transfer function phase shift.** The first 15 seconds are displayed. The entire signal comprised 3000 heart beats.
