# Supplementary material for: Cerebral autoregulation assessed by near-infrared spectroscopy: validation using transcranial Doppler in patients with controlled hypertension, cognitive impairment and controls
Source: Eur J Appl Physiol. 2021 Apr 16;121(8):2165–76. doi: 10.1007/s00421-021-04681-w (PMC8260523; doi:10.1007/s00421-021-04681-w)
Supplement: Supplementary file 2 — Supplementary file2 (DOCX 32 kb) [file 421_2021_4681_MOESM2_ESM.docx]

**Supplementary Table S2: Transfer function analysis results during supine rest and repeated sit-to-stand transitions**

|  |  | **Supine rest** | | | | | | | | | | | | |  | **Sit to stand transitions** | | |  |  |
| --- | --- | --- | --- | --- | --- | --- | --- | --- | --- | --- | --- | --- | --- | --- | --- | --- | --- | --- | --- | --- |
|  |  | **Younger controls (N=53)** | | | **Older controls (N=28)** | | | | **Patients with controlled hypertension (N=27)** | | **Cognitively impaired patients (N=94)** | | **All (N=202)** | |  | **Cognitively impaired patients (N = 94)** | | |  |  |
| **BP-CBFV** | | |  | | | |  |  | | | | | | | | | | |  |  |
| VLF | | | | | | | | | | | | | | | | | | |  |  |
|  | Gain, cm/s/mmHg, mean (SD) | 52 | | 0.55 (0.22) | 23 | 0.53 (0.14) | | | 25 | 0.45 (0.19) | 70 | 0.44 (0.15) | 170 | 0.49 (0.18) |  | 71 | 0.45 (0.24) | | |  |
|  | Phase, degrees, mean (SD) | 52 | | 50 (27) | 23 | 42 (14) | | | 25 | 48 (23) | 70 | 52 (29) | 170 | 50 (26) |  | 71 | 47 (13) | | |  |
|  | Coherence [0-1], mean (SD) | 53 | | 0.43 (0.18) | 23 | 0.54 (0.16) | | | 26 | 0.48 (0.21) | 72 | 0.53 (0.16) | 174 | 0.49 (0.18) |  | 71 | 0.81 (0.15) | | |  |
| LF | | | | | | | | | | | | | | | | | | |  |  |
|  | Gain, cm/s/mmHg, mean (SD) | 53 | | 0.91 (0.33) | 23 | 0.78 (0.23) | | | 26 | 0.63 (0.27) | 72 | 0.65 (0.20) | 174 | 0.74 (0.28) |  | 70 | | 0.53 (0.27) |  |  |
|  | Phase, degrees, mean (SD) | 53 | | 37 (16) | 23 | 30 (9) | | | 26 | 34 (10) | 72 | 31 (18) | 174 | 33 (16) |  | 70 | | 24 (13) |  |  |
|  | Coherence [0-1], mean (SD) | 53 | | 0.69 (0.17) | 23 | 0.69 (0.21) | | | 26 | 0.63 (0.22) | 72 | 0.63 (0.21) | 174 | 0.66 (0.20) |  | 71 | | 0.67 (0.18) |  |  |
| HF | | | | | | | | | | | | | | | | | | |  | |
|  | Gain, cm/s/mmHg, mean (SD) | 53 | | 1.04 (0.35) | 23 | 0.87 (0.26) | | | 26 | 0.67 (0.28) | 71 | 0.70 (0.28) | 173 | 0.82 (0.33) |  | 70 | | 0.43 (0.23) |  |  |
|  | Phase, degrees, mean (SD) | 53 | | 6 (15) | 23 | 5 (8) | | | 26 | 8 (11) | 71 | 1 (19) | 173 | 4 (16) |  | 70 | | 0 (14) |  |  |
|  | Coherence [0-1], mean (SD) | 53 | | 0.64 (0.20) | 23 | 0.67 (0.22) | | | 26 | 0.56 (0.27) | 72 | 0.63 (0.24) | 174 | 0.63 (0.23) |  | 71 | | 0.68 (0.21) |  |  |
| **BP-O_2_Hb** | | | | | | | | | | | | | | | | | | |  |  |
| VLF | | | | | | | | | | | | | | | | | | |  |  |
|  | Gain, cm/s/mmHg, mean (SD) | 44 | | 0.06 (0.03) | 20 | 0.10 (0.05) | | | 19 | 0.04 (0.01) | 67 | 0.05 (0.02) | 150 | 0.06 (0.03) |  | 40 | | 0.05 (0.03) |  |  |
|  | Phase, degrees, mean (SD) | 41 | | 72 (33) | 19 | 59 (19) | | | 18 | 60 (21) | 61 | 60 (25) | 139 | 63 (27) |  | 38 | | 44 (20) |  |  |
|  | Coherence [0-1], mean (SD) | 47 | | 0.39 (0.17) | 21 | 0.50 (0.23) | | | 23 | 0.40 (0.22) | 73 | 0.45 (0.18) | 164 | 0.43 (0.19) |  | 42 | | 0.71 (0.21) |  |  |
| LF | | | | | | | | | | | | | | | | | | |  |  |
|  | Gain, cm/s/mmHg, mean (SD) | 46 | | 0.06 (0.03) | 20 | 0.08 (0.04) | | | 23 | 0.04 (0.02) | 71 | 0.04 (0.02) | 160 | 0.05 (0.03) |  | 41 | | 0.04 (0.02) |  |  |
|  | Phase, degrees, mean (SD) | 41 | | 33 (31) | 19 | 17 (15) | | | 21 | 20 (27) | 64 | 12 (25) | 145 | 20 (27) |  | 39 | | 9 (26) |  |  |
|  | Coherence [0-1], mean (SD) | 47 | | 0.51 (0.22) | 21 | 0.46 (0.23) | | | 23 | 0.52 (0.26) | 73 | 0.44 (0.22) | 164 | 0.48 (0.23) |  | 42 | | 0.44 (0.14) |  |  |
| HF | | | | | | | | | | | | | | | | | | |  |  |
|  | Gain, cm/s/mmHg, mean (SD) | 46 | | 0.04 (0.02) | 21 | 0.05 (0.02) | | | 22 | 0.03 (0.01) | 73 | 0.02 (0.01) | 162 | 0.03 (0.02) |  | 42 | | 0.02 (0.01) |  |  |
|  | Phase, degrees, mean (SD) | 41 | | -0 (34) | 20 | 2 (33) | | | 21 | -0 (28) | 65 | 0 (31) | 147 | 0 (32) |  | 39 | | -3 (61) |  |  |
|  | Coherence [0-1], mean (SD) | 47 | | 0.43 (0.23) | 21 | 0.29 (0.19) | | | 23 | 0.45 (0.29) | 73 | 0.35 (0.20) | 164 | 0.38 (0.23) |  | 42 | | 0.34 (0.16) |  |  |

Results of BP-CBFV and BP-O_2_Hb transfer function analysis, expressed as mean gain, phase and coherence in the very low frequency (VLF), low frequency (LF) and high frequency (HF) range, per pooled cohort. SD: standard deviation.
