# Supplementary material for: Cerebral autoregulation assessed by near-infrared spectroscopy: validation using transcranial Doppler in patients with controlled hypertension, cognitive impairment and controls
Source: Eur J Appl Physiol. 2021 Apr 16;121(8):2165–76. doi: 10.1007/s00421-021-04681-w (PMC8260523; doi:10.1007/s00421-021-04681-w)
Supplement: Supplementary file 3 — Supplementary file3 (DOCX 14 kb) [file 421_2021_4681_MOESM3_ESM.docx]

**Supplementary Table S3: Cerebral autoregulation estimates derived from TCD and NIRS during supine rest, stratified by NIRS device**

|  |  | **NIRO-200NX**  **(N = 66)** | | **Portalite**  **(N = 17)** | | **Oxymon Mk III**  **(N = 112)** | |
| --- | --- | --- | --- | --- | --- | --- | --- |
| **BP-CBFV and BP-O_2_Hb** | | | | | | | |
| VLF | |  |  |  |  |  |  |
|  | Spearman correlation | 46 | 0.29 | 11 | -0.25 | 63 | 0.22* |
|  | BA bias (loa), degrees | 46 | 24 (126) | 11 | 2 (135) | 63 | 14 (112) |
| LF | |  |  |  |  |  |  |
|  | Spearman correlation | 44 | 0.27 | 9 | 0.24 | 58 | 0.30** |
|  | BA bias (loa), degrees | 44 | 2 (107) | 9 | -11 (66) | 58 | -5 (86) |

Spearman correlations and Bland Altman (BA) analysis results between NIRS- and TCD-derived CA measures within cohorts with similar NIRS device used. BP: blood pressure; CBFV: cerebral blood flow velocity; O_2_Hb: oxygenated hemoglobin; loa: separation between 95% upper and lower limits of agreement. One and two stars indicate statistically significant correlations with p values lower than 0.05 and 0.01, respectively.
